# Quasispecies of Hepatitis C Virus Participate in Cell-Specific Infectivity

**DOI:** 10.1038/srep45228

**Published:** 2017-03-22

**Authors:** Takasuke Fukuhara, Satomi Yamamoto, Chikako Ono, Shota Nakamura, Daisuke Motooka, Hiroyuki Mori, Takeshi Kurihara, Asuka Sato, Tomokazu Tamura, Takashi Motomura, Toru Okamoto, Michio Imamura, Toru Ikegami, Tomoharu Yoshizumi, Yuji Soejima, Yoshihiko Maehara, Kazuaki Chayama, Yoshiharu Matsuura

**Affiliations:** 1 Department of Molecular Virology, Research Institute for Microbial Diseases, Osaka University, Osaka, Japan; 2 Laboratory of Veterinary Microbiology, School of Veterinary Medicine, Kitasato University, Aomori, Japan; 3 Department of Infection Metagenomics, Research Institute for Microbial Diseases, Osaka University, Osaka, Japan; 4 Department of Surgery and Science, Graduate School of Medical Sciences, Kyushu University, Fukuoka, Japan; 5 Department of Gastroenterology and Metabolism, Applied Life Sciences, Institute of Biomedical & Health Sciences, Hiroshima University, Hiroshima 734-8551, Japan

## Abstract

It is well documented that a variety of viral quasispecies are found in the patients with chronic infection of hepatitis C virus (HCV). However, the significance of quasispecies in the specific infectivity to individual cell types remains unknown. In the present study, we analyzed the role of quasispecies of the genotype 2a clone, JFH1 (HCVcc), in specific infectivity to the hepatic cell lines, Huh7.5.1 and Hep3B. HCV RNA was electroporated into Huh7.5.1 cells and Hep3B/miR-122 cells expressing miR-122 at a high level. Then, we adapted the viruses to Huh7 and Hep3B/miR-122 cells by serial passages and termed the resulting viruses HCVcc/Huh7 and HCVcc/Hep3B, respectively. Interestingly, a higher viral load was obtained in the homologous combination of HCVcc/Huh7 in Huh7.5.1 cells or HCVcc/Hep3B in Hep3B/miR-122 cells compared with the heterologous combination. By using a reverse genetics system and deep sequence analysis, we identified several adaptive mutations involved in the high affinity for each cell line, suggesting that quasispecies of HCV participate in cell-specific infectivity.

More than 160 million individuals worldwide are infected with hepatitis C virus (HCV), and cirrhosis and hepatocellular carcinoma induced by HCV infection are life-threatening diseases^1^. Current standard therapy combining peg-interferon (IFN), ribavirin (RBV) and protease inhibitor has achieved a sustained virological response (SVR) in over 80% of patients infected with HCV genotype 1. In addition, many antiviral agents targeting non-structural proteins and host factors involved in HCV replication have been applied in a clinical setting^2^. On the other hand, re-infection of HCV in drug abusers or recipients of transplanted liver grafts remains a serious problem^3^,^4^.

With respect to primary HCV infection, HCV is naturally cleared in approximately 30% of cases. The major differences between primary infection and re-infection of a transplanted liver are as follows: 1) HCV quasispecies escaping from the immune response are already present in the serum at liver transplantation; 2) extra-hepatic HCV can serve as a reservoir for infection of the graft; and 3) the recipients must undergo immunosuppression after liver transplantation^3^. Several reports have demonstrated that quasispecies were dynamically changed immediately after liver transplantation^5^,^6^. However, there have been no reports about the mechanistic role of quasispecies in HCV adaptation to new target cells.

*In vitro* systems have been developed for the study of HCV infection and have revealed many details of the life cycle of HCV. By using pseudotype particles bearing HCV envelope proteins and RNA replicon systems, many host factors required for entry and RNA replication have been identified, respectively^7^,^8^. In addition, development of a robust *in vitro* propagation system of HCV based on the genotype 2a JFH1 strain (HCVcc) has gradually clarified the mechanism of the HCV life cycle^9^,^10^. Recently, several reports have shown that the expression of miR-122 in hepatic cancer cell lines facilitates the efficient propagation of HCVcc^11^,^12^. We reported that the efficiency of HCVcc propagation in Hep3B cells stably expressing miR-122 (Hep3B/miR-122) was comparable with that in Huh7 cells^11^.

In this study, the pattern of adaptive mutation and role of quasispecies in the infectivity of HCVcc were determined by using *in vitro* and *in vivo* models of HCVcc infection to hepatic cancer cell lines and uPA-SCID mice with human liver xenografts, respectively. The results suggested that quasispecies play crucial roles in the specific infectivity to new target cells.

## Results

### *In vitro* model for evaluating the role of quasispecies in the propagation of HCV

**Figure 1:**
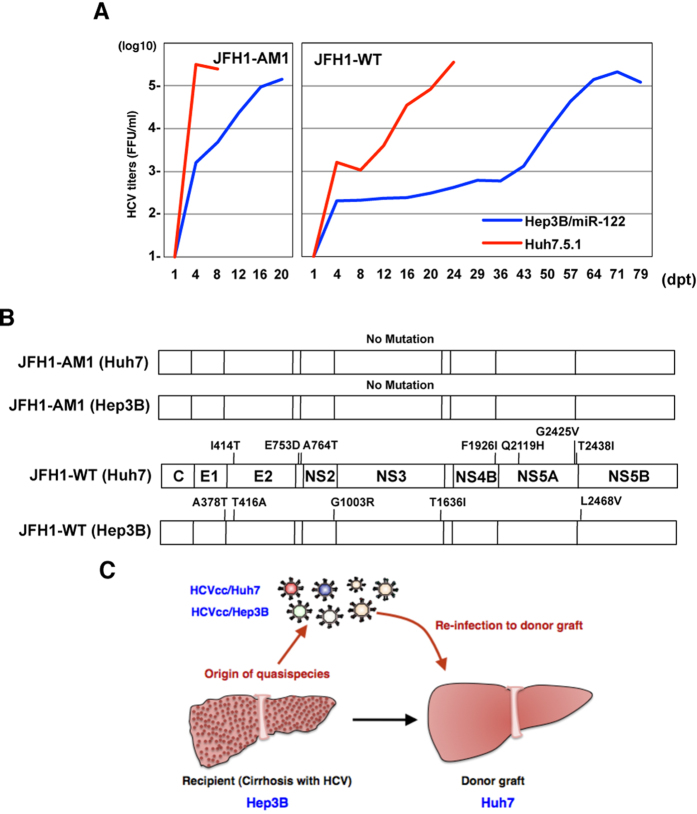
Production of Huh7- and Hep3B-adapted HCVcc. (**A**) *In vitro*-transcribed JFH1-AM (left panel) and JFH1-WT (right panel) RNA were electroporated into Huh7.5.1 (red line) and Hep3B/miR-122 (blue line) and infectious titers of each cell line were examined by focus forming assay. (**B**) Adaptive mutations of JFH1-AM and JFH1-WT in the supernatants of Huh7.5.1 and Hep3B/miR-122 cells. (**C**) Schematic diagram of adaptation of HCVcc to new target cells for the examination of the significance of quasispecies of HCV in infection to naive liver graft.

We previously reported that exogenous expression of miR-122 facilitates the efficient propagation of HCVcc in Hep3B cells^11^. Russell *et al*. also showed that the infectivity of HCVcc containing 3 mutations in the E2, p7 and NS2 proteins (JFH1-AM) was higher than that of parental HCVcc (JFH1-WT) in Huh7.5 cells^13^. In the present study, to obtain Huh7- and Hep3B-adapted HCVcc (HCVcc/Huh7 and HCVcc/Hep3B), we first electroporated *in vitro*-transcribed JFH1-AM and JFH1-WT RNA into Huh7.5.1 and Hep3B/miR-122 cells, then determined the titers in the supernatants at every serial passage by focus forming assay (Fig. 1A). Until 4 and 16 days post-transfection of JFH1-AM RNA, the infectious titers in the supernatants of both Huh7.5.1 and Hep3B/miR-122 cells increased up to 10^5^ FFU/ml. However, no adaptive mutations were observed in the viruses obtained at 20 days after electroporation, suggesting that the replication fitness of JFH1-AM was high enough not only in Huh7.5.1 but also in Hep3B/miR-122 cells (Fig. 1B). On the other hand, the infectious titer of JFH1-WT virus reached up to 200,000 FFU/ml, and several adaptive mutations emerged at 71 days post-transfection in Hep3B/miR-122 cells, indicating that the amino acid substitutions are essential for the adaptation of JFH1-WT. Also in Huh7.5.1 cells, 7 non-synonymous mutations emerged not in JFH1-AM but in the JFH-WT virus through serial passages. Therefore, we used JFH1-WT-based virus populations produced in Huh7.5.1 and Hep3B/miR-122 cells as Huh7- and Hep3B-adapted HCVcc (HCVcc/Huh7 and HCVcc/Hep3B), respectively. To identify the cell-specific adaptive mutation, we repeated the same experiments twice and compared the non-synonymous adaptive mutations between Huh7.5.1 and Hep3B/miR-122 cells. However, the cell-specific common adaptive mutations could not be identified, possibly due to a diverse emerging pattern of adaptive mutations. In this study, we used these 2 hepatic cancer cell lines and 2 adapted HCVcc viruses for analysis of the role of quasispecies in cell-specific infection of HCV (Fig. 1C).

### Adaptive mutation plays an important role in cell-specific replication fitness

**Figure 2:**
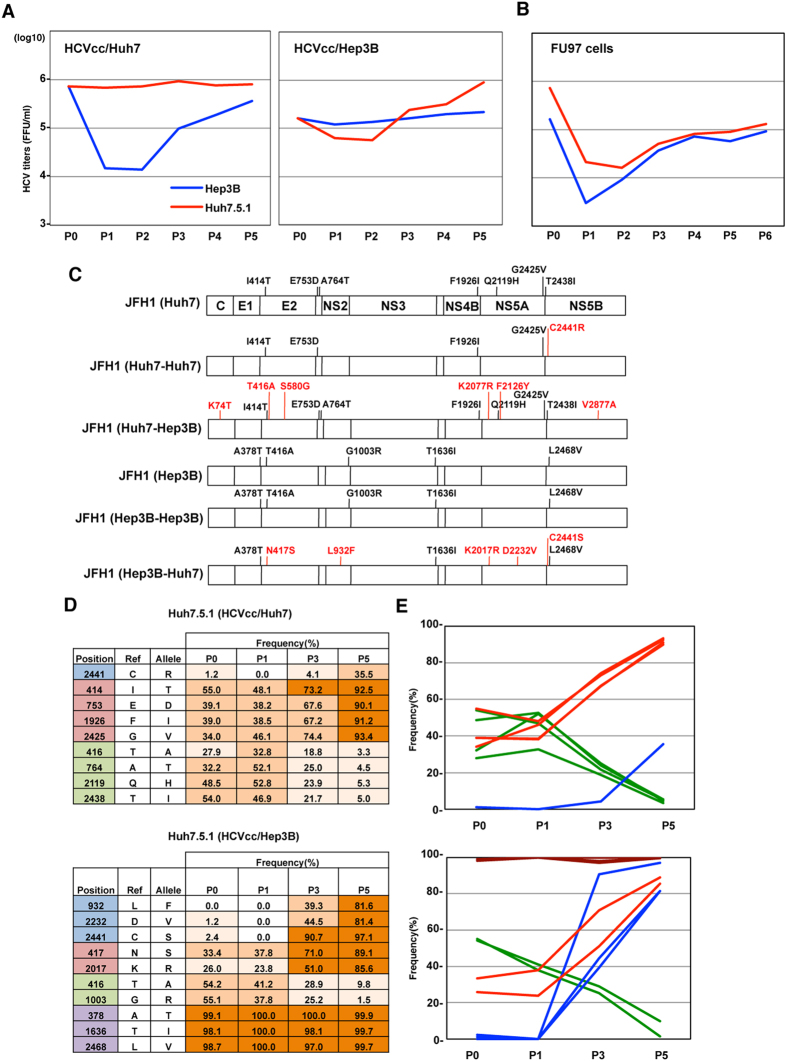
Change of genetic quasispecies is essential for adaptation to new target cells. (**A**) Infectious titers in the supernatants on serial passages of Hep3B/miR-122 and Huh7.5.1 cells infected with HCVcc/Huh7 (left panel) and HCVcc/Hep3B (right panel). (**B**) Infectious titers in the supernatants on serial passages of FU97 cells infected with HCVcc/Huh7 (red line) and HCVcc/Hep3B (blue line). (**C**) Distribution of mutations through the adaptation to Huh7.5.1 and/or Hep3B/miR-122 cells. Huh7- and/or Hep3B-adapted HCVcc were obtained through serial passages. Sequences were determined by direct sequencing and compared with JFH1-WT. (**D**) Frequency and position of mutations in HCVcc/Huh7 and HCVcc/Hep3B through serial passages in Huh7.5.1 cells. Produced viral particles in the supernatants were purified by ultracentrifugation, and total RNA was applied to deep sequence analysis. Frequencies of mutations in HCVcc/Huh7 and HCVcc/Hep3B were listed at every passage, and were classified into 4 patterns (am1-am4). (**E**) Changes in the frequencies of adaptive mutations are shown in the line graph.

To examine the change of replicative fitness in new host cells, we first measured the infectious titers in supernatants of Huh7.5.1 and Hep3B/miR-122 cells at 72 h post-infection with HCVcc/Huh7. Although the infectious titer of HCVcc/Huh7 in Hep3B cells was 70 times lower than that in Huh7.5.1 cells, the titers in Hep3B cells gradually increased over the 5 passages (Fig. 2A). Interestingly, the efficiency of propagation of HCVcc/Hep3B was also transiently decreased in Huh7.5.1 cells and increased through the serial passages. Recently, we identified a novel permissive cell line, FU97, which could be used for the complete propagation of HCVcc^14^. As we expected, transient decreases in the formation of infectious HCVcc/Huh7 and HCVcc/Hep3B particles were observed in FU97 cells (Fig. 2B). These results suggest that HCV recovers its high replication fitness through the adaptation to new host cells. Therefore, the sequences of HCVcc/Huh7 and HCVcc/Hep3B were determined after the adaptation to Hep3B/miR-122 and Huh7.5.1 cells, respectively (Fig. 2C). Through the adaptation to Hep3B/miR-122 and Huh7.5.1 cells, an additional 6 and 5 mutations were observed in HCVcc/Huh7 and HCVcc/Hep3B, respectively. On the other hand, only one additional mutation was identified in HCVcc/Huh7 over the 5 passages, suggesting that the emergence of the adaptive mutations and selection of quasispecies occurred in a dynamic manner for the adaptation to new host cells. To further examine the change of quasispecies in the adaptation, the frequency of genetic variants of HCV was determined by deep sequence analysis (Fig. 2D and E). To lessen the bias by PCR-mediated amplification of the HCV genome, the produced viral particles in supernatants were purified by ultracentrifugation, and total RNA of the purified virus was directly applied to deep sequence analysis. The frequency of genetic variants of HCV except for the 5′ and 3′ untranslated regions (UTRs) could be identified by deep sequence analysis, and the frequencies of adaptive nonsynonymous mutations through 5 passages in Huh7.5.1 cells are listed in Fig. 2D. As in Fig. 2C, 8 and 7 nonsynonymous mutations were identified in HCVcc/Huh7 and HCVcc/Hep3B, respectively. Through 5 passages in Huh7.5.1 cells, the frequency of 4 nonsynonymous mutations of HCVcc/Huh7 increased up to 90%. On the other hand, the frequency of mutations in HCVcc/Hep3B changed in diverse ways through the adaptation to Huh7.5.1 cells, and the adaptive mutations in HCVcc/Hep3B were classified into 4 groups per the pattern of change of frequency (am1 to am4). In HCVcc/Hep3B, am1 from the wild-type sequence was almost replaced, and am2 and am3 co-existed within the wild-type sequence. Although am2 and am4 were selected through the adaptation to Huh7.5.1 cells, the frequency of am3 was decreased. These results suggest that a dynamic change of quasispecies is involved in the adaptation of HCV to naive target cells. Considering the similar transitional change of mutation frequency in each group, the mutations combined in the same HCV genome were quickly selected due to the difference of replication fitness to naive target cells.

**Figure 3:**
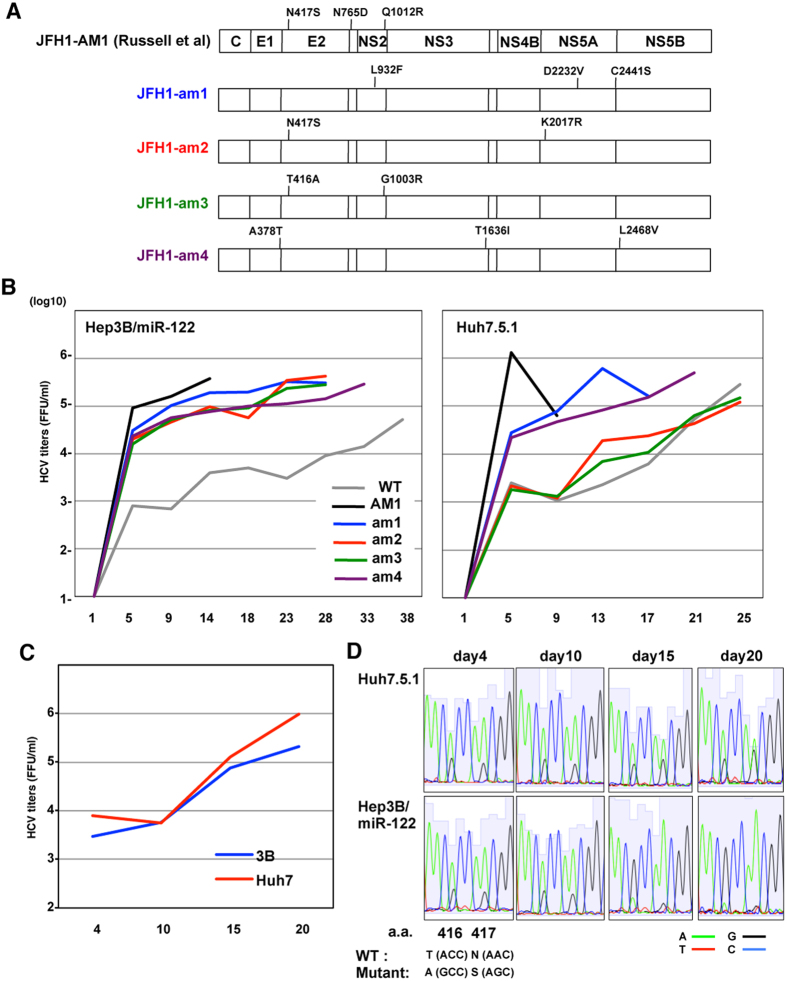
Serial virus passages induce cell-specific adaptive mutations for high replicative fitness. (**A**) Distribution of mutations in JFH1-am1, -am2, -am3 and -am4. (**B**) *In vitro*-transcribed viral RNAs of JFH1-WT (grey), -AM (black), -am1 (blue), -am2 (red), -am3 (green) and -am4 (purple) were electroporated into Hep3B/miR-122 and Huh7.5.1 cells and infectious titers in the supernatants were determined at several points by focus forming assay. (**C**,**D**) Equivalent amounts of JFH1-WT, -am2 and -am3 RNA were co-electroporated into Hep3B/miR-122 (blue line) and Huh7.5.1 cells (red line), and infectious titers (**C**) and the sequences at the positions of amino acids 416 and 417 (**D**) in the supernatants were determined at 4, 10, 15 and 20 days post-electroporation by focus forming assay and direct sequencing, respectively.

To further confirm the effect of adaptive mutation on cell-specific infectivity, we electroporated *in vitro*-transcribed HCV RNA containing each adaptive mutation (am1-am4: Fig. 3A) into Hep3B/miR-122 and Huh7.5.1 cells, and analyzed the infectious titers serially. The titers of JFH1-am1 and JFH1-am4 were quickly elevated both in Hep3B/miR-122 and Huh7.5.1 cells, compared to those of JFH1-WT. On the other hand, the titers of JFH1-am2 and JFH1-am3 were comparable with those of JFH1-WT in Huh7.5.1 cells, but higher than those of JFH1-WT in Hep3B cells. These results suggest that JFH1-am2 and JFH1-am3 have high replication fidelity only in Hep3B/miR-122 cells. To compare the replication fidelity of these mutants in Huh7.5.1 and Hep3B/miR-122 cells, identical amounts of JFH1-WT, JFH1-am2 and JFH-am3 RNA were co-electroporated, and the time course of infectious titers and frequency of mutants were determined (Fig. 3C and D). Both in Huh7.5.1 and Hep3B/miR-122 cells, the infectious titers were over 10^5^ FFU/ml until 20 days post-electroporation. Although all mutants were maintained until 20 days post-electroporation in Huh7.5.1 cells, only JFH1-am3 was selected through 4 passages in Hep3B/miR-122 cells. These results indicate that this adaptive mutation enhances the infectivity of HCV in a cell-specific manner.

### Adaptive mutations facilitate efficient propagation of HCV in new target cells

**Figure 4:**
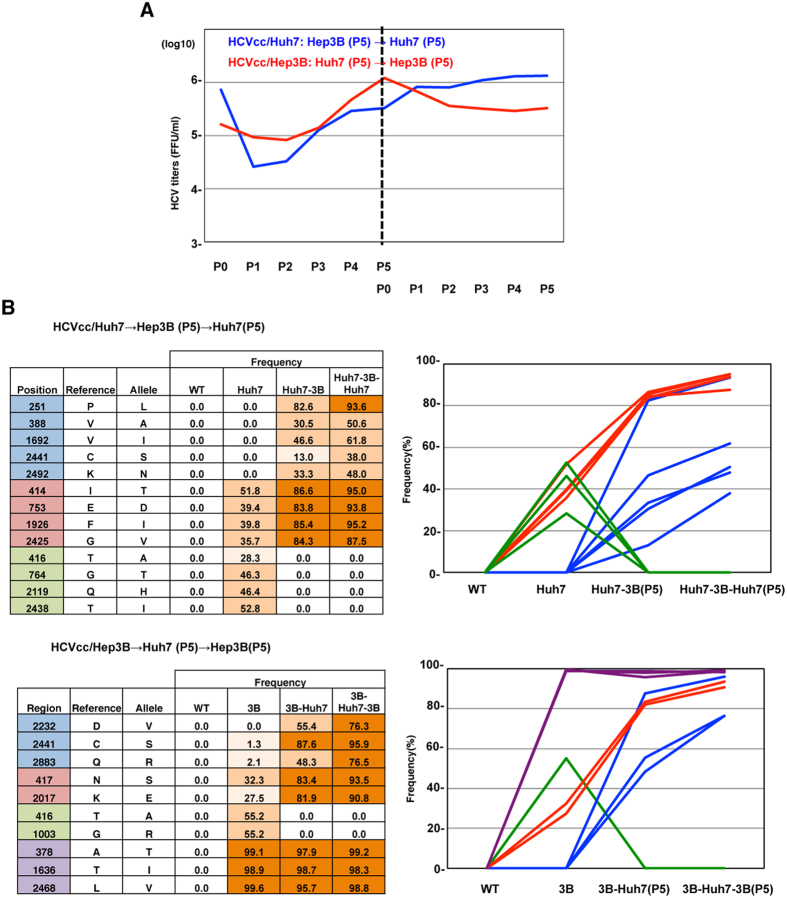
Adaptive mutations facilitate efficient propagation of HCV in new target cells. (**A**) Infectious titers in the supernatants on serial passages of Hep3B/miR-122 and Huh7.5.1 cells infected with HCVcc/Huh7 (left panel) and HCVcc/Hep3B (right panel). After the adaptation to Hep3B/miR-122 and Huh7.5.1 cells, HCVcc/Huh7 and HCVcc/Hep3B were used for serial infection of Huh7.5.1 and Hep3B/miR-122 cells, and infectious titers in the supernatants were determined by focus forming assay. (**B**) The frequency and position of mutations in HCVcc/Huh7 and HCVcc/Hep3B through serial passages both in Huh7.5.1 and Hep3B/miR-122 cells were determined by deep sequencing. (**C**) Changes in the frequencies of adaptive mutations are shown in the line graph.

To examine whether adaptive mutants observed both in Huh7.5.1 and Hep3B/miR-122 cells emerged over several passages, the time course of infectious titers and frequencies of adaptive mutations of HCVcc/Huh7 and HCVcc/Hep3B were determined after 5 passages both in Hep3B/miR-122 and Huh7.5.1 cells, respectively. Although a transient decrease of infectious titers was observed through the adaptation to new target cells, HCVcc/Huh7 and HCVcc/Hep3B maintained a high replication fidelity in Huh7.5.1 and Hep3B/miR-122 cells even after the adaptation to Hep3B/miR-122 and huh7.5.1 cells, respectively (Fig. 4A). Both in HCVcc/Huh7 and HCVcc/Hep3B infection, adaptive mutations emerged through the adaptation to Hep3B/miR-122 and Huh7.5.1 cells, respectively, as shown by the blue lines in Fig. 4B. However, no additional mutations in HCVcc/Huh7 and HCVcc/Hep3B emerged through the passages in Huh7.5.1 and Hep3B cells after the adaptation to both cell lines. These results suggest that the emergence and selection of adaptive mutations are essential for efficient propagation in new target cells.

### A different HCV variant was selected in uPA-SCID mice bearing transplanted human liver cells

**Figure 5:**
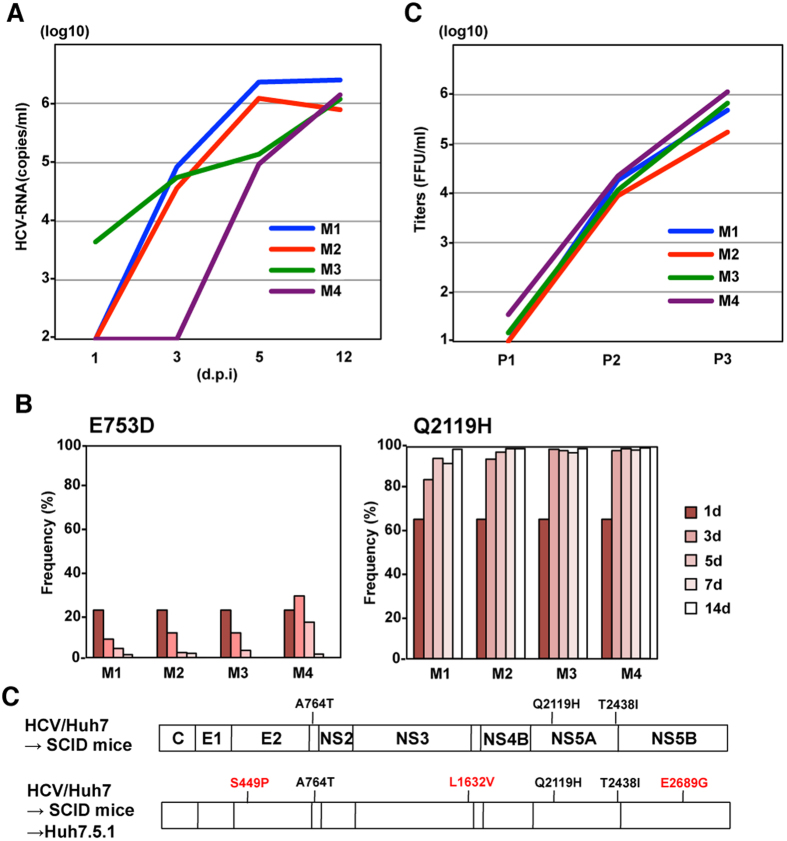
A different HCV variant was selected in chimeric mice with human liver xenografts. (**A**) Viral RNA levels in the sera of chimeric mice with human liver were determined by qRT-PCR at 1, 3, 5 and 12 days post-infection with HCVcc/Huh7. (**B**) Viral sequences including E753D and Q2119H were amplified by PCR and frequencies of mutations were evaluated by deep sequencing. (**C**) Distribution of mutations in chimeric mice-adapted and Huh7-re-adapted HCV. (**D**) Infectious titers in the supernatants on serial passages of Huh7.5.1 cells infected with HCV derived from chimeric mice at 12 days post-infection.

To examine the change of quasispecies *in vivo*, HCVcc/Huh7 and HCVcc/Hep3B were administered to human liver-transplanted chimeric mice (n = 4 mice in total), and viral RNA levels were determined by qRT-PCR. Unfortunately, infection of HCVcc/Hep3B was established only in 1 mouse, presumably due to the low replication fitness of these viruses compared to HCVcc/Huh7 infection (Fig. 5A). Therefore, the frequencies of 2 pre-existing substitutions of HCVcc/Huh7 in chimeric mice were determined by deep sequence analysis in order to examine the change of quasispecies (Fig. 5B). Although E753 mutants were quickly diminished by 14 days post-infection, the frequency of Q2119H mutants was increased to over 98% in all mice. In addition, sequence analysis of the full-length genome of HCV adapted to chimeric mice revealed that 3 pre-existing mutations in HCVcc/Huh7 were selected (Fig. 5C), suggesting that a transmission bottleneck of HCVcc/Huh7 was strongly induced in the chimeric mice with a human liver xenograft. To examine the replicative fitness of uPA-SCID mouse-derived HCV to hepatic cancer cells, the infectivity and sequence of the viral genome in Huh7.5.1 cells infected with mouse-derived HCV were determined (Fig. 5C and D). Through serial passages, the infectious titers of these viruses in supernatants increased up to 10^5^ FFU/ml and several adaptive mutations emerged, suggesting that the high replicative fitness of HCVcc/Huh7 to Huh7 cells was lost by transmission bottleneck in chimeric mice.

## Discussion

RNA viruses naturally generate variants due to replication in the absence of proofreading-repair activities in RNA-dependent RNA polymerases that replicate these viruses. Consequently, an RNA virus such as HCV replicates and circulates in a patient as a population of genomes named quasispecies, composed of a complex mixture of different but closely related genomes^15^ that undergoes continuous changes due to competitive selection and cooperation^16^ between arising mutants. The variants of the hepatic viruses that are continuously generated may have clinical relevance since they may affect pathogenesis, induce peripheral immune tolerance, escape from vaccination or mediate resistance to antiviral therapy^17^,^18^,^19^,^20^,^21^,^22^,^23^,^24^,^25^,^26^. In the present study, we show detailed genomic changes in HCV quasispecies that facilitate their adaptation and increase their replicative fitness features in two cell lines permissive to HCVcc infection.

Many adaptive mutations of HCV that permit the virus to propagate efficiently in Huh7 or Huh7.5.1 cells have been identified in previous reports^13^,^27^,^28^,^29^,^30^. Mutations in E2, p7 and NS2 have been particularly well-associated with such adaptation. In the current analyses, introduction of these 3 mutations (JFH1-AM1) strongly enhanced replication fitness both in Huh7 and Hep3B cells, facilitating efficient propagation without emergence of other mutations. Also in HCVcc/Huh7 or HCVcc/Hep3B, several mutations in E2, p7 and NS2 were observed, and were shown to be involved in cell-specific adaptation (Fig. 3A and B). Although several mutations in structural and non-structural proteins were observed through the adaptation, it is difficult to determine whether such adaptive mutations were combined within the same genome. However, in some of the adaptive mutations, frequency rate was similar each other through the serial passages, and thery could be regarded as a group of the mutations (Figs 2D and 4B), indicating that the observed adaptive mutations existed in the same HCV genome.

During the transmission process in both acute and chronic infection of HCV, the quasispecies are dynamically changed by environmental changes such as host immune response. HCV genomic diversity is modulated by the bottleneck at viral transmission through blood transfusion or liver transplantation, and different genome populations are formed during the acute and chronic phases of HCV infection. The previous reports have shown that the evolution of quasispecies and escape from selective pressure of the host immune system are involved in persistence and clearance during the acute phase of HCV infection^31^,^32^. Considering the correlation between IL28B genotype and the spontaneous clearance of HCV, the bottleneck of quasispecies might be affected by differences in such host factors^33^. Also in the current study, cancer cell-adapted HCV quasispecies were quickly selected in chimeric mice with a human liver xenograft, and replicative fitness to hepatic cell lines was decreased. These results suggest that HCV quasispecies are dynamically selected and changed, leading to the acquisition of high replicative fitness in different environments. In fact, competition between the HCV genomic evolution and the restrictions imposed by the host, including immune responses, has been reported. Ray *et al*. suggested that HVR1 may act as an immunologic decoy during the acute phase of HCV infection, facilitating viral persistence^34^. Long-term analyses of the change of quasispecies in chronic hepatitis C patients and chimpanzees have revealed that HCV maintains a balance between replicative fitness and immune escape from cytotoxic T lymphocytes (CTLs) and neutralizing antibody within the context of chronic infection^35^,^36^,^37^. In addition, a recent report showed that immunoregulatory changes during pregnancy reduce the selective pressure to HCV quasispecies and emergence of more fit viruses, facilitating vertical transmission to the child^38^.

In the current study, transient decreases in the infectivity and in the change of viral populations were observed for adaptation to different cell lines upon infection with HCVcc/Huh7 and HCVcc/Hep3B in Hep3B and Huh7 cells, respectively. In addition, a transmission bottleneck of HCV populations was observed in chimeric mice with a human liver xenograft. Despite the transmission bottleneck of the viral genome and low fidelity RdRp-mediated emergence of new populations in subsequent hosts, such varied populations of viral genome are essential for adaptability to different environments. Several reports have shown that high replicative error of poliovirus is important for the survival of viral populations and a mutant with increased fidelity is attenuated in mice, suggesting that quasispecies participate in viral transmission^39^. Borucki *et al*. demonstrated that low replication fidelity of rabies virus was essential for the efficient cross-species transmission between skunks and foxes^40^. In addition, varied populations induced by low fidelity of RdRp of the chikungunya virus facilitate trans-infection between vertebrates and invertebrates^41^. Because HCV exhibits a narrow host range, infecting only humans and chimpanzees, the origin or evolution of HCV has remained to be elucidated^42^. Recently, deep sequence analyses have revealed that chronic infection of HCV-related hepacivirus is observed in the livers of several animals^43^. Therefore, establishment of a novel infection model of HCV-related hepaciviruses in non-primate cells might facilitate investigation of the evolution of hepacivirus during cross-species transmission.

In conclusion, the current data suggest that quasispecies of HCV participate in the efficient transmission and acquisition of high fitness to different environments. Further studies will be needed to identify adaptive mutants with high fidelity and clarify the significance of HCV quasispecies in replicative fitness. In addition, the establishment of an *in vivo* HCV-induced pathogenesis model and hepacivirus infection model might lead to understanding about the association of quasispecies with pathogenesis and viral evolution, respectively.

## Methods

### Analysis of quasispecies of HCV-RNA (PCR-SSCP, cloning, and direct sequencing)

For reverse transcription and nested PCR, Superscript 3 First-Strand Synthesis SuperMix (Invitrogen, Tokyo, Japan) and TaKaRa Ex-Taq (Takara Bio Inc, Shiga, Japan) were used. The E2 region including the hypervariable region 1 (HVR-1) was amplified as previously described^5^. PCR products were purified and were resuspended in 20 μL of water, and 5 μL of PCR product was mixed with 5 μL of loading buffer, heated for 2** **min at 98 °C, and rapidly cooled on ice. For analysis, 6 μL of the diluted PCR product was loaded onto a GeneGel SSCP gel (GE Healthcare Bio-Sciences KK, Tokyo, Japan) and electrophoresed at 600 V for 2 h at a constant temperature of 5 °C. The bands were visualized by silver staining. Ethidium bromide gel-purified PCR product was ligated into a T7-Blue vector for TA-cloning and used to transform competent Escherichia coli JM109, then cultured on an LB agar plate with ampicillin. Twenty colonies were selected and plasmid DNA was purified using QIAprep mini kit (QIAGEN, Tokyo, Japan). The sequences of the HVR-1 were determined by direct sequencing using a BigDye Terminator v3.1 Cycle Sequencing Kit and ABI3130 Genetic Analyzer (Applied Biosystems Inc., Japan). Quasispecies analysis was performed using the MEGA program. The genetic diversity, defined as the frequency of mutations within different HCV quasispecies, was expressed in terms of genetic distance, where the genetic distance of the quasispecies was estimated by pairwise comparison of all amino acid sequences using the p-distance method.

### Cell lines

All cell lines were cultured at 37 °C under the conditions of a humidified atmosphere and 5% CO_2_, and maintained in DMEM (Sigma) supplemented with 100 U/ml penicillin, 100 μg/ml streptomycin, and 10% fetal bovine serum (FBS). The human hepatocellular carcinoma-derived Huh7 and human embryonic kidney-derived 293 T cells were obtained from the Japanese Collection of Research Bioresources (JCRB) Cell Bank (#JCRB0403 and JCRB9068). The Huh7-derived cell line Huh7.5.1 was kindly provided by F. Chisari^44^. Hep3B cells were obtained from the American Type Culture Collection (ATCC).

### Plasmids

The cDNA clones of pri-miR-122 were inserted between the XhoI and XbaI sites of lentiviral vector pCSII-EF-RfA, which was kindly provided by M. Hijikata, and the resulting plasmids were designated pCSII-EF-miR-122. The plasmid pJFH1, which encodes the full-length cDNA of the JFH1 strain, was kindly provided by T. Wakita. pJFH1-AM contains three adaptive mutations in pJFH1^13^. The plasmids used in this study were confirmed by sequencing with an ABI 3130 genetic analyzer (Thermo Fisher Scientific).

### Antibodies

Rabbit anti-NS5A antibody was prepared as described previousl^45^. Alexa Fluor (AF) 488-conjugated anti-rabbit IgG antibody was purchased from Thermo Fisher Scientific.

### Preparation of viruses

The plasmids pJFH1 and pJFH1-AM were linearized with XbaI, and treated with mung bean exonuclease. The linearized DNA was transcribed *in vitro* by using a MEGAscript T7 kit (Thermo Fisher Scientific) according to the manufacturer’s protocol. The *in vitro*-transcribed JFH1 and JFH1-AM RNA were introduced into Huh7.5.1 cells, HCVcc in the supernatant was collected after serial passages, and infectious titers were determined by a focus-forming assay and expressed in focus-forming units (FFU). All mouse studies were conducted at Hiroshima University (Hiroshima, Japan) in accordance with the guidelines of the local committee for animal experiments. Chimeric mice transplanted with human hepatocytes were generated. The experimental protocol was approved by the Ethics Review Committee for Animal Experimentation of the Graduate School of Biomedical Sciences (Hiroshima University). The chimeric mice were infected with a 4 × 10^5^ titer of HCVcc.

### Lipofection and lentiviral gene transduction

The lentiviral vectors and ViraPower Lentiviral Packaging Mix (Thermo Fisher Scientific) were co-transfected into 293 T cells by Trans IT LT-1 (Mirus), and the supernatants were recovered at 48 h post-transfection. The lentivirus titer was determined by using a Lenti-XTM qRT-PCR Titration Kit (Clontech), and the expression levels and AcGFP were determined at 48 h post-inoculation.

### Immunoblotting

Cells lysed on ice in lysis buffer (20 mM Tris-HCl [pH7.4], 135 mM NaCl, 1% Triton-X 100, 10% glycerol) supplemented with a protease inhibitor mix (Nacalai Tesque) were boiled in loading buffer and subjected to 5–20% gradient SDS-PAGE. The proteins were transferred to polyvinylidene difluoride membranes (Millipore) and reacted with the appropriate antibodies. The immune complexes were visualized with SuperSignal West Femto Substrate (Pierce) and detected by an LAS-3000 image analyzer system (Fujifilm).

### Quantitative RT-PCR

Total RNA was extracted from cells by using an RNeasy minikit (Qiagen), and the first-strand cDNA synthesis and qRT-PCR were performed with TaqMan EZ RT-PCR core reagents and a ViiA7 system (Thermo Fisher Scientific), respectively, according to the manufacturer’s protocol. The primers for TaqMan PCR targeted to the noncoding region of HCV RNA were synthesized as previously reported^45^. Fluorescent signals were analyzed with the ViiA7 system.

### Deep Sequencing of HCV-RNA

Culture supernatants of cells infected with HCVcc were concentrated 50 times by using Spin-X UF concentrators (Corning), and total RNA was extracted from purified HCV particles or serum from chimeric mice with human liver xenografts and reverse transcribed to complementary DNA (cDNA) using an RNeasy minikit and PrimeScript RT reagent kit. The Illumina sequencing library was prepared from 1.5 ng of cDNA using a Nextera XT DNA Sample Prep Kit (Illumina). Paired-end sequencing was performed with 300 cycles on a MiSeq sequencer (Illumina). Sequence read mapping and variant calling were conducted using the CLC genomics workbench (QIAGEN).

### Statistics

The data for statistical analyses are the average of three independent experiments. Results were expressed as the means ± standard deviation. The significance of differences in the means was determined by Student’s *t*-test.

## Additional Information

**How to cite this article:** Fukuhara, T. *et al*. Quasispecies of Hepatitis C Virus Participate in Cell-Specific Infectivity. *Sci. Rep.* **7**, 45228; doi: 10.1038/srep45228 (2017).

**Publisher's note:** Springer Nature remains neutral with regard to jurisdictional claims in published maps and institutional affiliations.
